# Pulmonary Function and Respiratory Muscle Strength in Patients with Multiple Sclerosis

**DOI:** 10.1155/2021/5532776

**Published:** 2021-06-14

**Authors:** Elisabeth Westerdahl, Martin Gunnarsson, Anna Wittrin, Ylva Nilsagård

**Affiliations:** ^1^Department of Physiotherapy, Faculty of Medicine and Health, Örebro University, SE 701 82 Örebro, Sweden; ^2^University Health Care Research Center, Faculty of Medicine and Health, Örebro University, SE 701 82 Örebro, Sweden; ^3^Department of Neurology, Faculty of Medicine and Health, Örebro University, SE 701 82 Örebro, Sweden

## Abstract

**Background:**

In patients with multiple sclerosis (MS), there is a decline in muscle strength and physical capacity due to demyelination and axonal loss in the central nervous system. In patients with advanced MS or in a later stage of the disease, also respiratory impairment may occur. The degree of pulmonary dysfunction in the earlier stages of MS has not been thoroughly described. Therefore, the primary aims of this study are to describe pulmonary function and respiratory muscle strength in patients with a moderate disease course and to identify associations between respiratory muscle strength and functional capacity.

**Methods:**

A sample of 48 patients with a diagnosis of MS and mean age 56 ± 11 years was studied using a descriptive cross-sectional design. The patients had a disease duration of 24 ± 11 years and a median Expanded Disability Status Scale (EDSS) score of 4.5 (interquartile range 4.0-6.5). Pulmonary function assessed by spirometry, respiratory muscle strength, peak cough flow and peripheral oxygen saturation, subjective breathing and coughing ability, and physical capacity measured using the 6MWT were evaluated.

**Results:**

The patients had normal pulmonary function with no significant abnormalities in dynamic spirometry (vital capacity 103 ± 16% predicted, forced expiratory volume in 1 second 95 ± 15% predicted). Peak expiratory flow rate 89 ± 17% predicted was in the lower limit of normal. Respiratory muscle strength, determined by maximal inspiratory (MIP) and expiratory (MEP) static pressures, was normal but with large differences between individuals. MIP ranged from 26 to 143 cmH_2_O (98 ± 31% predicted); the MEP values ranged from 43 to 166 cmH_2_O (104 ± 29% predicted), with two patients having values below the lower limit of normal. Significant positive associations between MIP as well as MEP were found in several pulmonary function variables. A significant negative association was found between EDSS score and MEP (*r* = −0.312, *p* = 0.031). Mean peak cough flow was 389 ± 70 L/min, which is comparable with the values reported for healthy adults. The patients did not experience a severely decreased ability to take deep breaths or cough. There was a moderate correlation between MEP and physical capacity, as assessed by the 6MWT (*r* = 0.399, *p* = 0.010) and between peak expiratory flow (PEF) and the 6MWT (*r* = 0.311, *p* = 0.048).

**Conclusion:**

Respiratory muscle strength, pulmonary function assessed by spirometry, and peak cough flow were normal in patients with mild to moderate MS; however, there were large individual differences demonstrating low respiratory muscle strength in some patients. Significant associations between MEP and functional capacity and between MEP and disease severity were found, indicating that patients with impaired respiratory muscle strength have lower functional capacity and more severe disease.

## 1. Introduction

In multiple sclerosis (MS), demyelination and axonal loss in the central nervous system may result in muscle weakness and physical impairment [1]. Respiratory muscle weakness can produce a restrictive breathing pattern on spirometry and may eventually lead to ventilatory failure. Respiratory complications are common in the terminal stages of the disease, and a decrease in pulmonary function and expiratory muscle strength has been reported in patients with advanced MS and markedly reduced mobility, i.e., bedridden or wheelchair-bound individuals [2, 3]. Expiratory muscle strength has been reported to be more affected than inspiratory muscle strength [2, 4], with a risk of impaired cough ability [5]. Morbidity and mortality are often caused by pulmonary complications such as aspiration pneumonia, atelectasis, or acute respiratory deficiency [6].

Even in patients with minor neurological deficits, respiratory muscle function has been found to be abnormal in two studies performed more than two decades ago [7–9] and in a more recent study in 14 MS patients by Altintas et al. [9]. Small reductions in pulmonary function, measured by spirometry [4] and diffusing capacity [9], have been reported in 38 patients with an Expanded Disability Status Scale (EDSS) score < 6.5, i.e., mild to moderate MS. None of these studies evaluated cough ability or physical outcomes. The sample sizes in the studies were small, and the degree of pulmonary dysfunction in the early stages of MS has not been thoroughly described.

When assessing suspected respiratory muscle weakness, knowledge about functional capacity can be useful, since the reduction in exercise tolerance may be related to respiratory muscle dysfunction [10, 11]. Walking distance is often measured in clinical practice and research, using standardized tests. The 6-minute walk test (6MWT) measures anaerobic and aerobic activity and correlates well with patient-reported limitations of walking capacity in MS [12, 13]. Associations between pulmonary function and walking capacity in patients with MS have been scarcely evaluated [11].

The primary aims of this study are to describe pulmonary function and respiratory muscle strength in patients with a mild to moderate disease course of MS and to identify associations between respiratory muscle strength and walking distance covered during 6 minutes. A secondary aim was to evaluate whether disease severity in MS is associated with pulmonary impairment. Our hypothesis was that reduced respiratory muscle strength and pulmonary function are correlated with limited physical capacity and that patients with more pronounced neurological deficits, as assessed by EDSS, have more marked pulmonary impairment.

## 2. Methods

### 2.1. Study Population

In this descriptive, cross-sectional study, a sample of 48 patients with MS, registered in the Swedish MS Register [14] and living in Örebro County, was selected from a previous investigation [15]. All patients aged 18 years or older with mild or moderate MS, according to the revised McDonald criteria [16], were eligible for inclusion. Inclusion criteria were relapse-free for at least 3 months prior to inclusion, able to understand verbal and written study information, and able to walk with or without the use of walking devices. Patients who had other neurological diseases, severe ischaemic heart disease, orthopaedic conditions, and language or cognitive difficulties which could adversely influence the performance of walking or pulmonary function tests were not included. The study was approved by the Regional Ethical Review Board in Uppsala, Sweden (2012/077), and informed consent was obtained from each patient. The main trial was registered at ClinicalTrials.gov (NCT01774201).

### 2.2. Outcome Measurements

Measurements were performed at Örebro University Hospital, Örebro, Sweden. The patients' current EDSS score was determined by a neurologist. The EDSS score ranges between 0 and 10, where 0 = no impairment, 4 = onset of significant walking impairment, 6 = onset of use of an assistive device during ambulation, and 10 = death due to MS [17, 18]. Demographic data (Table 1) were collected from the Swedish MS registry and from the neurologist. A written questionnaire about subjective breathing and coughing ability was completed by the patient. A physiotherapist experienced in neurology assessed pulmonary function based on spirometry and peripheral oxygen saturation (SpO_2_). Respiratory muscle strength test and 6MWT were then performed, and the overall health status was assessed by EQ-5D visual analogue scale.

#### 2.2.1. Pulmonary Function Assessed by Spirometry

Pulmonary function variables were measured by spirometry using a MicroLab spirometer (MicroMedical/CareFusion, Kent, UK). The equipment was calibrated every morning prior to measurements. The spirometry was performed with patients in both a sitting and lying position. A nose clip was used, and measurements were performed as recommended by the American Thoracic Society (ATS)/European Respiratory Society (ERS) [19]. The highest value of three technically satisfactory manoeuvres was retained. Slow vital capacity (VC) was obtained during an inspiratory manoeuvre, followed by measurement of forced vital capacity (FVC), forced expiratory volume in one second (FEV_1_), peak expiratory flow (PEF), and FEV% (FEV1/VC_max_). Predicted values for pulmonary function were related to age, sex, and height [20].

#### 2.2.2. Respiratory Muscle Strength

Measurement of maximal static pressure for determination of respiratory muscle strength was performed from total lung capacity for maximal expiratory pressure (MEP) and from residual volume for maximal inspiratory pressure (MIP), as described in the ATS/ERS Statement on Respiratory Muscle Testing [21].

Maximum respiratory pressures were measured using a microrespiratory pressure meter (RPM) (MicroMedical/CareFusion, Kent, UK). Measurements were performed in a sitting position and with a nose clip. Patients were instructed to press their lips tightly against the flanged mouthpiece and to support their cheeks manually during the manoeuvre, to prevent air leaks. The highest value from at least five technically acceptable attempts was recorded and expressed as an absolute value (cmH_2_O) and as a percentage of the predicted value (% predicted) adjusted for age and sex [22].

#### 2.2.3. Peak Cough Flow

A portable peak flow meter (Vitalograph, Ennis, Ireland) connected to a face mask was used to measure coughing ability assessed as peak cough flow (PCF). The patients were asked to take a deep breath outside the mask and then put the mask tightly on the face and cough as much as possible with the mask on. The best value of three attempts was noted.

#### 2.2.4. Peripheral Oxygen Saturation

Peripheral oxygen saturation (SpO_2_) was measured using a pulse oximeter device (Rad-5v; Masimo, Irvine, CA, USA).

#### 2.2.5. Subjective Breathing and Coughing Ability

All patients scored their perceived breathing and coughing ability on a numeric rating scale (NRS), from 0 (“no difficulty”) to 10 (“impossible”); they scored their level of dyspnea while walking indoors and while climbing stairs on a numeric rating scale (NRS) from 0 (“no dyspnea”) to 10 (“worst imaginable dyspnea”).

#### 2.2.6. Physical Capacity

Physical capacity was assessed using the 6MWT, which measures the distance covered in 6 minutes. A physiotherapist instructed the patient to walk as far as possible at their maximum for 6 minutes. The patients were encouraged to walk as fast as they could back and forth in a quiet hallway in a 30 m straight line. No encouragement was given during the test procedure, and rest was not allowed [13]. The use of a walking aid, if needed, was recorded. The outcome was distance walked. The 6MWT is a reliable and valid submaximal test for assessing functional capacity in patients with functional impairments.

### 2.3. Statistical Analysis

Data are presented as mean ± standard deviation (SD) or median (md) and interquartile range (IQR) or expressed as absolute and relative frequencies (%). Pearson's correlation test was used for associations between respiratory muscle strength and spirometry and between EDSS and respiratory muscle strength or spirometry. Two-tailed *p* values ≤ 0.05 were considered statistically significant. Version 25.0 of the SPSS software package (SPSS Inc., Chicago, IL, USA) was used for the statistical analysis.

## 3. Results

In total, 48 patients, aged 22–73 years, primarily with mild or moderate symptoms of MS and with sustained walking ability were studied. Characteristics of the study sample are presented in Table 1. The distribution of patients among the different subtypes of MS was as follows: 42% relapsing-remitting multiple sclerosis (RRMS), 54% secondary progressive multiple sclerosis (SPMS), and 4% primary progressive multiple sclerosis (PPMS). The median EDSS score was 4.5 (IQR 4.0-6.5). Patients rated their overall health status on the EuroQol Five Dimension (EQ-5D) visual analogue scale (VAS) from 0 = worst imaginable to 100 = best imaginable health as 65 ± 22 (range 10–100), md (25–75% IQR) 68 (50–80).

### 3.1. Pulmonary Function Assessed by Spirometry

The mean pulmonary function was normal for the sample (*n* = 48), as related to the reference values; VC 103 ± 16% predicted. FEV1, 95 ± 15%, was within the normal range, although PEF, 89 ± 17% predicted, was in the lower limit. Four of the patients were defined as having airway obstruction (FEV_1_/VC_max_) < 0.70. Spirometry results are presented in Table 2. No correlation was found between disease severity (EDSS) and pulmonary function variables (VC (*r* = −0.037, *p* = 0.802), FVC (*r* = −0.052, *p* = 0.728), and FEV1 (*r* = −0.011, *p* = 0.941)).

### 3.2. Respiratory Muscle Strength

Maximal static pressures for determination of respiratory muscle strength were normal on a group level but with large individual differences (Table 2). The MIP values ranged from 26 to 143 (mean 80 ± 28) cmH_2_O (98 ± 31% predicted). The MEP values ranged from 43 to 166 (mean 97 ± 25) cmH_2_O (104 ± 29% predicted), with two patients reaching below the lower limit of normal, according to reference values [22]. Significant positive associations between respiratory muscle strength and pulmonary function (VC, FVC, FEV1, and PEF) were found as presented in Table 3. A significant negative association was also found between disease severity (EDSS) and MEP (*r* = −0.312, *p* = 0.031) but not for MIP (*r* = −0.097, *p* = 0.511).

### 3.3. Peak Cough Flow

The mean value for PCF was 389 ± 70 (range 240-570) L/min, which is comparable with the values reported for healthy adults [23]. A significant association was found between MIP and PCF (*r* = 0.397, *p* = 0.005) as well as MEP and PCF (*r* = 0.437, *p* = 0.002). No association was found between disease severity (EDSS score) and PCF (*r* = −0.249, *p* = 0.087).

### 3.4. Peripheral Oxygen Saturation

The SpO_2_ was 97 ± 2% (range 92–100%), with 13 patients having SpO_2_ < 96%.

### 3.5. Subjective Breathing and Coughing Ability

The patients experienced no severe decreased ability to take deep breaths; NRS 0; range 0–5 or cough; NRS 0; range 0–6. Dyspnea was reported to be scarce while walking indoors (0; 0–6) and while climbing stairs (0; 0–8). The mean values for subjective breathing and coughing ability are presented in Table 4.

### 3.6. Physical Capacity

Physical capacity was assessed using the 6MWT, and the mean walking distance was 312 ± 138 m (*n* = 41). There was a moderate correlation between MEP and the distance covered during the 6MWT (*r* = 0.399, *p* = 0.010) and between PEF and the 6MWT (*r* = 0.311, *p* = 0.048).

## 4. Discussion

The main finding of the present study is that ambulatory patients with mild to moderate disease course of MS displayed normal pulmonary function as measured by dynamic spirometry. Mean values in both MIP and MEP were also in accordance with normal ranges but with large individual differences. A significant negative association was found between disease severity (EDSS score) and MEP but not MIP, and this is in line with previous results [24].

Although there were normal respiratory muscle strengths, there were large differences between patients, indicating lower respiratory muscle strength in some patients. The normal values for MIP and MEP have a large range, and there are several reference equations available in the literature. In the present study, six patients had lower MIP values than 50 cmH_2_O, considered to be sufficient for normal breathing according to Evans et al. [22]. MEP more than 60 cmH_2_O is necessary to produce an effective cough, and in our sample, three patients had lower values than this [22].

Impaired expiratory muscle strength in patients with MS has been described in early studies published decades ago [8, 10], and marked expiratory muscle weakness has been demonstrated in bedridden and wheelchair-bound patients [25]. Previous studies in MS patients with mild to moderate symptoms (EDSS 1 to 6.5) have shown varying results regarding respiratory impairments in ambulatory patients. Savci et al. [26] demonstrated that pulmonary function and respiratory muscle strength of ambulatory MS patients were significantly lower than healthy controls. Also, walking distance measured as 6MWT was reduced but was not explained by the lung function impairment. Ray et al. [27] showed normal pulmonary function in patients with but slightly reduced MIP, MEP, and 6MWT compared to population standards.

An inverse correlation between expiratory muscle strength and disease severity has been described in patients with pronounced neurological deficits [25] as well as in patients with mild to moderate MS (EDSS 1.5–6.5) [4, 5, 7], which is in accordance with our results.

In the present study, respiratory muscle strength was found to be normal compared with reference values [22]. Similarly as in healthy persons [28], large individual differences between patients were found in our study; MIP values ranged from 26 cmH_2_O to 143 cmH_2_O and MEP values from 43 to166 cmH_2_O. Maximal static pressures measured at the mouth, i.e., MIP and MEP, were used to assess inspiratory and expiratory muscle strength as recommended [21, 29]. In patients with good collaboration and coordination, MIP and MEP represent the best physiological assessment of respiratory muscle strength. The main limitation of these measurements relates to the difficulty to perform breathing manoeuvres correctly, and this can be especially difficult for some patients. The validity of these measurements depends on voluntary effort, patient motivation, and careful understanding of the instructions. To increase reliability, we used the highest value from at least five technically acceptable attempts, as recommended.

In previous studies, correlations between impaired pulmonary function, assessed by spirometry, and EDSS scores were found in MS patients with a range of motor impairments [4, 8, 25], but in the present study, no such correlations were found. However, a correlation was found between respiratory muscle strength and spirometry values, although it should be noted that VC and its subdivisions are fairly insensitive indicators of respiratory muscle weakness. Respiratory muscle strength has to be reduced to a large extent before VC is changed, and pulmonary function may be preserved even if respiratory muscle function is weakened [10]. It is known that a reduction of MIP and MEP has been recognized in several neuromuscular diseases. Significantly reduced pulmonary function indicating impaired cough efficacy has previously been described in severely disabled and wheelchair-bound MS patients [2] but was not seen in our patients with less severe disability. Expiratory muscle strength and the ability to cough can easily be assessed by measuring PEF and PCF, and these assessments may be useful for monitoring expiratory muscle weakness and for assessing its evolution in these patients [30].

During normal breathing, the inspiratory work is mostly dependent on the diaphragm function, and the accessory respiratory muscles become necessary only during deeper inspiration. The patient group showed a lower PEF (89% predicted) compared with normal values, although FEV1 was within the normal range. The mean value for PCF was 389 ± 70 L/min, and there was a significant association between PCF and both MIP and MEP. Effective removal of airway secretions depends on expiratory flow, i.e., PCF. According to previous studies, a PCF value of at least 160 L/min is needed for effective removal of airway secretions [31]. However, no correlation between EDSS and PCF was found, so indicators other than disease severity are needed for further pulmonary function evaluation. A larger sample size is moreover necessary to detect less substantial associations.

Limited walking ability is common in MS, especially in progressive disease. Physical capacity was assessed using the 6MWT, and the mean walking distance was 312 ± 138 m. In this study, we did not detect any association between pulmonary function and walking ability; however, there were significant associations between MEP and 6MWT and between MEP and disease severity, indicating that patients with low MEP also have low functional capacity and more severe disease. This is an important result, which should be spread to clinicians and persons with MS. The respiratory muscles are used for many purposes other than respiration, and their strength is not determined solely by requirements for breathing. Even if MIP and MEP are decreased, the strength may still be well above the level for maintenance of normal breathing and the capacity required to sustain respiration at rest.

There are several limitations in the present study. The sample was collected from a previous randomized controlled trial [15], but considering that all data for the present study was collected at baseline, i.e., before intervention started, the randomization should not affect the results. There is limited covariates/confounders in the statistical analysis, and the sample size is small (*n* = 48), which limits the generalizability of the results. Further limitations in the present study was the exclusion of patients who had severe ischaemic heart disease, orthopaedic conditions, or cognitive difficulties which could adversely influence the performance of walking tests. It is important to notice that our results cannot be generalized to these patient groups. The predicted values for both MIP and MEP decrease with age, and the elastic recoil is affected by smoking which could have been valuable to study in more detail; unfortunately, we had not data for pack-year available.

The patient group experienced no severe subjective impairment of the ability to take deep breaths or to cough. Dyspnea was reported to be rare while walking indoors or while climbing stairs; however, this might reflect that factors other than breathing difficulties are limiting the physical ability. Impaired walking capacity in MS is mainly a consequence of motor or sensory deficits in the lower extremities, as well as discoordination or balance disturbances. However, other factors such as reduced respiratory muscle strength may contribute to limitations in exercise at an individual level.

Recent studies suggest that the expiratory muscles in MS patients can be trained for strength and endurance [2, 15, 32, 33]. The optimal timing of the introduction of these breathing exercises in the course of the MS disease is not known, and there is today no evidence to recommend specific respiratory rehabilitation programmes [34].

Dynamic spirometry, MIP, MEP, and PCF are reliable outcome measures that can be assessed at bedside. A large individual variation in respiratory muscle strength was found in this sample of mildly to moderately disabled MS patients. Further studies are needed to study pulmonary function in more critically ill MS patients and to examine whether evaluation of pulmonary and respiratory muscle function could be useful, based on the level of disability, for the management of patients in a longer and preventive perspective. Especially assessment during exercise could be emphasized. By adding simple measures, MEP test, and 6MWT for patients' annual follow-up in patients with mild and moderate disease severity, the possibility to identify patients at risk would increase. Early interventions to improve expiratory muscle strength could possibly improve functional capacity but that needs further to be evaluated.

## 5. Conclusions

Pulmonary function, assessed by dynamic spirometry, and respiratory muscle strength were in this study found to be normal in patients with mild to moderate MS; however, there were large individual differences indicating low respiratory muscle strength in some patients.

Significant associations were found between MEP and functional capacity and between MEP and disease severity, indicating that patients with more pronounced respiratory muscle strength impairment have lower functional capacity and more severe disease.

## Figures and Tables

**Table 1:** Characteristics of the sample (*n* = 48).

Male/female, *n*	13/35
Age, years	56 ± 11
BMI, kg/m^2^	27 ± 4
Never smoked/ex-smoker/smoker, *n*	21/17/10
Pack year	15 ± 11 [2–38]
Cough: never/occasionally/often	36/9/3
Secretion problems: never/occasionally/often	41/5/2
Airway obstruction^∗^, *n*	4
MS disease duration, years	24 ± 11
Relapsing-remitting MS, *n*	20 (42%)
Secondary progressive MS, *n*	26 (54%)
Primary progressive MS, *n*	2 (4%)
EDSS, median (25–75% IQR)	4.5 (4.0–6.5)
Cognitive dysfunction/PASAT, *n*	22 (46%)
Exacerbation during the last 3 months, *n*	2 (4%)
Physiotherapy treatment ongoing, *n*	23 (48%)
Walking aids, unilateral or bilateral, *n*	15 (31%)
Wheelchair use, *n*	2 (4%)

^∗^Patients with forced expiratory volume in 1 second/maximal vital capacity (FEV_1_/VC_max_) < 0.70 were defined as having airway obstruction. Data are presented as mean ± standard deviation (SD), [min-max], or number (*n*) and percentage (%) of patients. BMI: body mass index; EDSS: Expanded Disability Status Scale; IQR: interquartile range; MS: multiple sclerosis; PASAT: Paced Auditory Serial Addition Test.

**Table 2:** Pulmonary function assessed by spirometry and respiratory muscle strength.

	Absolute values	Percentage of predicted values
VC (L)	3.5 ± 0.9	103% ± 16%
FVC (L)	3.5 ± 1.0	103% ± 15%
FEV1 (L)	2.7 ± 0.7	95% ± 15%
FEV_1_/VC (%)	76.5 ± 7.0	98% ± 9%
PEF (L/min)	369 ± 89	89% ± 17%
MIP, cmH_2_O	80 ± 28, 81 [60-92]	98% ± 31%
MEP, cmH_2_O	97 ± 25, 96 [79-96]	104% ± 29%

Data are presented as mean ± standard deviation (SD), median [interquartile range], and as percentage of predicted values (*n* = 48). FEV_1_: forced expiratory volume in 1 second; FVC: forced vital capacity; MEP: maximal expiratory pressure; MIP: maximal inspiratory pressure; PEF: peak expiratory flow; VC: vital capacity.

**Table 3:** Correlation between MIP/MEP and spirometry (*n* = 48).

	MIP *r*	*p* value	MEP *r*	*p* value
VC (L)	0.517	0.000	0.344	0.017
FVC (L)	0.509	0.000	0.298	0.040
FEV1 (L)	0.523	0.000	0.307	0.034
FEV_1_/VC (%)	0.000	0.99	0.001	0.996
PEF (L/min)	0.655	0.000	0.507	0.000

FEV_1_: forced expiratory volume in 1 second; FVC: forced vital capacity; MEP: maximal expiratory pressure; MIP: maximal inspiratory pressure; PEF: peak expiratory flow; VC: vital capacity.

**Table 4:** Subjective breathing and coughing ability.

Ability to take deep breaths	0.5 ± 1.2 (0–5)
Ability to cough	0.8 ± 1.7 (0–8)
Dyspnea while walking outside	0.3 ± 0.7 (0–3)
Dyspnea while climbing stairs	1.4 ± 1.6 (0–5)

Data are presented as mean ± standard deviation (SD) (*n* = 48).

## Data Availability

Readers may contact the authors for access to the deidentified dataset.
